# Recurrent apical prolapse after high uterosacral ligament suspension – in a heterogenous cohort characterised by a high prevalence of previous pelvic operations

**DOI:** 10.1186/s12905-019-0800-8

**Published:** 2019-07-12

**Authors:** Katrine Dahl Pedersen, Marie Højriis Storkholm, Karl Møller Bek, Marianne Glavind-Kristensen, Susanne Greisen

**Affiliations:** 1 Department of Gynaecology and Obstetrics, Aarhus University Hospital, Palle Juul-Jensens Boulevard 99, 8200 Aarhus N, Denmark; 2 Department of Gynaecology and Obstetrics, Regional Hospital in Horsens, Horsens, Denmark; 3 Brabrand, Denmark

**Keywords:** Vaginal vault prolapse, Uterosacral ligament suspension, Recurrent prolapse, Pelvic organ prolapse, Surgery

## Abstract

**Background:**

The apical prolapse is probably the most complex form of pelvic organ prolapse (POP). Adequate apical support is essential in the treatment of POP, as it contributes to the support in all vaginal compartments. This study aimed to evaluate the rate of symptomatic recurrent apical prolapse after high uterosacral ligament suspension (HUSLS), in a cohort of women characterised by a high prevalence of previous pelvic operations and a significant degree of prolapse.

**Methods:**

This is a retrospective chart review of 95 women who underwent HUSLS for symptomatic apical prolapse from 2002 to 2009 at Aarhus University Hospital, Denmark. Of these women, 97% attended a six-month clinical control. Recurrence was defined as symptomatic vaginal vault prolapse stage 2 or more (according to the International Continence Society (ICS) quantification system). Medical charts were reviewed for a mean period of 7.2 years. Any new contacts due to prolapse were noted.

**Results:**

Before the operation, 73% of the women were hysterectomised, and 52% had previous prolapse surgery. Stage 2 apical prolapse was reported in 71% of the women, whereas 26% had stage 3 or 4.

At six-month follow-up, 19% of the women had recurrent symptomatic apical prolapse, and 9% of the women had symptomatic recurrent prolapse in other compartments 6 months after operation. In all, 35% of the women had a renewed prolapse operation during the long-term follow-up period.

Perioperative adverse events were seen in 7%.

Two women were re-operated due to postoperative complications.

**Conclusions:**

This retrospective study of 95 women with a significant degree of prolapse and a high prevalence of previous pelvic operations demonstrates that the rate of recurrent prolapse associated with HUSLS might be higher than originally described. In conclusion, HUSLS may not be the optimal first choice of operation in this group of patients.

## Background

Pelvic organ prolapse (POP) is a common health problem, and its incidence is increasing due to ageing populations as well as increasing obesity rates [1]. POP may occur in up to 50% of parous women [2]; however, the majority of these women are asymptomatic.

POP is associated with physical, psychological, and sexual problems, as well as a negative influence on the quality of life amongst the affected women [1]. Lifetime risk of surgery for prolapse or urinary incontinence is 11% at the age of 80. Approximately 29% of these women require a second surgery [3].

The apical prolapse is probably the most complex form of POP. Adequate apical support is essential in the treatment of POP, as it contributes to the support in all vaginal compartments. Anterior and posterior vaginal repairs may be unsuccessful if the apex is not well suspended [2, 4].

There are a variety of surgical treatments for vaginal apical prolapse. Transvaginal performance of the procedure occurs in 80–90% of cases [2, 4].

In 2000, Shull et al. [5] described an operation with high uterosacral ligament suspension (HUSLS), and this operation has since become a common method for apical repair. Shull et al. found that 87% of the 289 women operated had an optimal anatomic outcome. Only 5% had grade 2 (Baden-Walker scale) or greater persistent or recurrent support defects in any compartment, and only two patients (0.7%) had repeat surgery.

Maher [4] previously reviewed the surgical treatment for apical prolapse; however, there currently is no consensus regarding the treatment of post-hysterectomy or recurrent apical prolapse [6]. In reccent years, there has been increased focus on potential complications associated with mesh use for POP repair, and several permanent mesh products have been withdrawn from the market by the manufacturers [7]. Therefore, interest in native-tissue repair procedures, such as HUSLS, has been renewed.

This study aims primarily to evaluate the rate of symptomatic recurrent apical prolapse after HUSLS in a cohort of women characterised by a high prevalence of previous operations for POP and a severe degree of POP.

## Methods

### Study design and patients

This study is a retrospective chart review of 95 women who underwent HUSLS for apical prolapse from January 2002 through 2009 at Aarhus University Hospital, Denmark.

The patients were identified by the procedural code (KLEF53). Prior to the operation, objective uterovaginal prolapse or vaginal vault prolapse was defined and clinically staged according to recommendations from the International Continence Society quantification system [8].

### Data collection

Medical charts were reviewed, and demographics, medical and surgical history, and intraoperative and postoperative data were collected.

All patients had transvaginal colpopexy with bilateral fixation of the vaginal vault to the uterosacral ligament. They were all invited to a follow-up visit and examination 6 months after surgery. Furthermore, medical charts were reviewed for an extended period – a minimum of 4 years – after surgery, and mean chart follow-up was 7.2 years. Any new contacts due to prolapse symptoms were noted. Since the department at Aarhus University Hospital is the sole department in the Central Region of Denmark to perform operations for vaginal vault prolapse, the women were unlikely to be referred elsewhere when presenting with recurrence.

Recurrent prolapse was defined as symptomatic prolapse stage 2 or greater of the vaginal vault, meaning that the most distal portion of the vaginal vault is between 1 cm proximal and 1 cm distal to the plane of the hymen [8].

### Data

Data are presented as an average (including standard deviation), ‘number of women’, and frequencies (%). A chi-squared test was performed and showed no significant differences in any of the preoperative parameters when comparing the women who had a recurrence of vaginal vault prolapse with the group of women who did not. We had no missing data.

### Surgical technique

All operations were performed ad modum Bob Shull [5] by two specialists in urogynaecology, at least one of them was very experienced in the procedure.

The operation was performed in general anaesthesia. All patients received a single dose of antibiotics intraoperatively. Patients underwent vaginal hysterectomy if they had not previously had this operation, most often an anterior and posterior repair was also performed, concomitant procedures are listed in Table 1. The peritoneum was opened, the small bowel was packed away from the operative field with a long gauze mesh, and a retractor positioned to expose the uterosacral ligaments. The ligaments were then bilaterally transfixed in their intermediate position at the level of or above the ischial spine, with three polydioxanone 0 sutures. Plication of the pubocervical and the rectovaginal fascia was performed with polydioxanone 3–0 sutures. The sutures from the uterosacral ligament were fixated to the apex of both the pubocervical and the rectovaginal fascia. The ligament sutures were tightened to close the vaginal cuff. Vaginal mucosa was trimmed and closed with a running polyglactin 3–0 suture. A diagnostic cystoscopy was performed at the end of the operation to assess ureteral bilateral patency. After the operation, the vagina was packed, and the patient had an indwelling catheter. The catheter and the vaginal packing were removed the morning after the operation.

**Table 1:** Description of the study population

	Total *N* = 95
Age ^a^	63 (SD 9,7 years)
BMI ^a^	25 (SD 4)
ASA ^b^	
1	46 (48%)
2	44 (46%)
3	5 (5%)
4	0 (0%)
Hysterectomy prior to surgery ^b^	69 (73%)
Previous pelvic floor surgery ^b^	
0:	46 (48%)
1:	30 (32%)
2–3:	19 (20%)
Diabetes ^b^:	5 (5%)
Smokers ^b^:	13 (14%)
Stage of apical prolapse prior to surgery ^b^	
0:	0 (0%)
1:	3 (3%)
2:	67 (71%)
3:	12 (13%)
4:	13 (14%)
Concomitant procedures ^b^	
Anterior and posterior repair	70 (74%)
Anterior repair	21 (22%)
Posterior repair	4 (4%)
Site of recurrence ^b^	
Vaginal vault (multi compartment)	18 (19%)
Posterior compartment	9 (9%)
Anterior compartment	4 (4%)
Anterior and posterior compartment.	4 (4%)
Repeat surgery	33 (35%)

^a^Mean *SD* Standard Deviation, ^b^ Number of women (%)

## Results

**Fig. 1:**
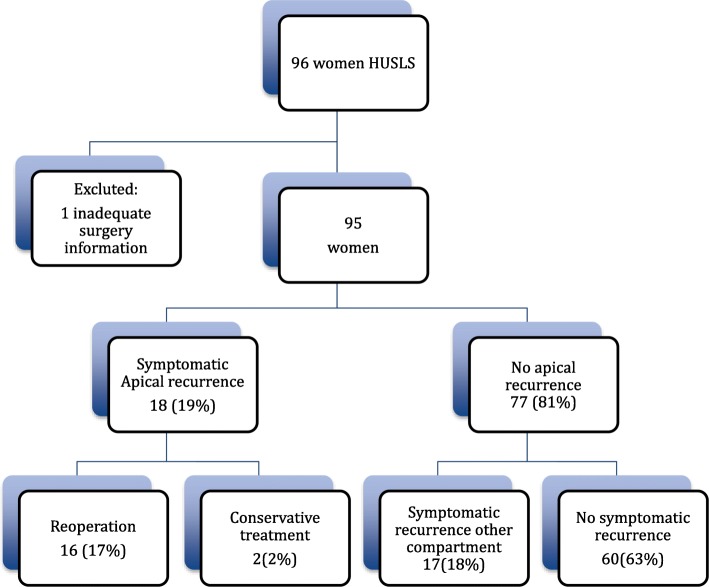
Flowchart of study population, exclusion, recurrence, and treatment

We identified 96 women who underwent HUSLS at Aarhus University Hospital from 2002 through 2009. One woman was excluded due to insufficient information about the surgery performed. The analysis included 95 women; a flow chart is presented in Fig. 1.

Ninety-two (97%) women attended the six-month follow-up. Three (3%) women, all asymptomatic, refrained from physical examination and were followed-up by phone.

The majority of the women, 71%, had grade 2 prolapse of the apical compartment before the surgery, whereas 26% had grade 3 or 4 prolapse. In all, 73% had a previous hysterectomy and 52% previous prolapse surgery. Only 16% had no previous pelvic operations. Patient characteristics are summarised in Table 1.

### Recurrence and repeat surgery

At six-month follow-up, 18 (19%) of the women had recurrence of symptomatic vaginal vault prolapse. During the extended follow-up, no additional women were referred with recurrent symptomatic apical prolapse.

Repeat surgery was performed in 16 (17%) of the cases. One woman declined repeat surgery, and in one case, a second surgery was unattractive due to a chronic pain disorder not related to the former operation.

Another nine women (9%) had symptomatic recurrent prolapse that did not involve the apical compartment at their six-month follow-up. This number rose to 17 women (18%) at the end of the long-term follow-up (Table 1). All of these women had repeat surgery.

In total, 27 (28%) of the women had recurrent prolapse at the six-month follow up. At the end of the long-term follow-up, a total of 33 (35%) women had had repeat surgery.

When comparing the women who had a recurrence of vaginal vault prolapse with the group of women who did not, there was no significant difference found in age, BMI, smoking habits, or any of the other preoperative parameters.

### Adverse events

The overall intraoperative adverse event rate was 7% (7/95). The intraoperative ureteral injury rate was 1% (1/95); bladder injury rate was 3% (3/95), and small intestine injury rate was 2% (2/95). One patient (1%) suffered an intraoperative haemorrhage of more than 200 ml.

The overall postoperative adverse event rate was 17% (16/95). Minor complications, such as postoperative urinary tract infection or short-term urinary retention, occurred in 15% (14/95) of the cases. However, 2% (2/95) of the women suffered from more complex postoperative complications. One woman was diagnosed with ureteral obstruction, hematoma, and infection. Another woman had a second surgery because of haemorrhage. Adverse events are summarised in Table 2.

**Table 2:** Adverse events

	Total 95 (number of women (%))
Perioperative adverse events total	7 (7%)
Bladder injury	3 (3%)
Ureteral injury/kinking	1 (1%)
Injury to the small intestine	2 (2%)
Hemorrhage (> 200 ml)	1 (1%)
Postoperative adverse events total	17 (18%)
Urinary retention	9 (10%)
Urinary tract infection	5 (5%)
Other ^a^	2 (2%)

^a^One woman: reoperation because of Bleeding

One woman: Hematoma and infection, this woman also had a postoperative ureteral obstruction

## Discussion

This retrospective study of 95 women operated for apical vaginal prolapse with HUSLS demonstrates that 19% of the women had a recurrence of symptomatic vaginal vault prolapse at a six-month follow-up. At this time, 9% had symptomatic recurrent prolapse that did not involve the apical compartment. A total of 35% had repeat surgery within the long-term follow-up period (mean follow-up 7.2 years).

The interest in native-tissue prolapse repair has been renewed due to its low cost and lack of mesh-related complications [9].

HUSLS is a well-described, native-tissue technique for treating apical prolapse. It is considered safe and effective; however, the rate of recurrence and reoperation has been reported as considerably different in various studies.

The modification of the HUSLS operation used in this study is originally described by Shull et al. [5]. In 302 patients operated by a single trained surgeon, 87% had an optimal anatomic outcome. Prolapse recurred in 13%, most frequently in the anterior segment, but only 5% had grade 2 (Baden-Walker scale) or greater prolapse in any compartment, and only two (0.7%) patients had repeated surgery.

Other authors have shown success rates comparable to Shull et al. (Barber et al. [10], Karram et al. [11], and R. Milani et al. [9]). A systematic review from 2010 found the anatomic success rates to be 98.3% for the apical compartment [12]. Only 9.4% of the women received a reoperation due to stress incontinence or symptomatic prolapse in any compartment. Minimum follow-up in the 10 articles included was 12 months. The number of prior prolapse operations was not reported.

Several new studies have not been able to demonstrate the same high success rate.

In the OPTIMAL randomised trial from 2014, uterosacral ligament suspension was compared to sacrospinous ligament suspension for treatment of apical prolapse [13]. Surgical success was defined by composite outcomes, including anatomic results, symptoms, and any retreatment. The surgical success rate two years after uterosacral ligament fixation was 59.2%.

In all, 188 women underwent HUSLS; 25.5% had prior hysterectomy, and 4.8% had prior prolapse surgery. Recurrence beyond the hymen was noted in 24.5% of the women; 19.2% had symptoms, but only 3.1% received a second surgery.Edenfield AL et al. conducted a retrospective cohort study published in 2013 [14]. Of the 219 women who were included, 7% had a prior prolapse surgery. The overall recurrent prolapse rate after a minimum of 6 months was 24.7%, including 8.7% with apical recurrence. Reoperation was performed in 15.1% of the women [14].

A large retrospective study by Unger et al. examined recurrent POP after transvaginal uterosacral colpopexy and laparoscopic and robotic sacral colpopexy [15]. They included 983 women who underwent uterosacral colpopexy; 7% of them had prior prolapse surgery. The estimated recurrence rate for uterosacral colpopexy was as high as 43% 6 years after the surgery [15].

Our data, like other studies [13, 15], cannot confirm the high surgical success rate found by Shull et al. [5]. Several things might explain the difference in success rates. Our study is based on a complex prolapse population with a high degree of prolapse and a high proportion having had previous prolapse operations. Such conditions are known to be important factors influencing surgical results [16–18].

In the cohort described by Shull et al., 32% of the patients had stage 0 or 1 prolapse (Baden-Walker scale). In our study population, only 3% of the women had stage 1 prolapse (ICSQ), and none of the women had stage 0.

Shull et al. reported that 70% of the women in their study had a previous hysterectomy, and 45% had prior pelvic reconstructive surgery. This is comparable to our cohort in which 73% had a previous hysterectomy and 52% previous prolapse surgery. However, only 5% of the women in Shull’s cohort had more than one previous prolapse operation, whereas 20% in our population had more than one previous prolapse operation.

Another difference between the studies is the fact that Shull himself operated on all 302 women included in his study. In our study, the operations were performed by experienced urogynaecologists; however, seven different surgeons conducted the 95 operations. We cannot, from this study, conclude that fewer surgeries per surgeon results in higher rates of recurrence and complications; however; it may be part of the explanation of the difference in success rates.

Another hypothesis might be that the choice of suture material influences the recurrence rate. Shull et al. used permanent sutures, whereas we used long-term absorbable sutures. Others have used both permanent and absorbable sutures [13, 19]. Bradley MS et al. [20] and Unger CA et al. [19] have addressed this issue; however, none of them found any difference in recurrence of apical prolapse when retrospectively comparing women operated with absorbable or permanent sutures.

Comparing reoperation rates after prolapse surgery is difficult since the treatment of women with recurrence is dependent on many factors. Of the women in our study with a recurrence of symptomatic prolapse, 94% received a second surgery. In the study by Shull et al., 38 women (13%) had recurrent prolapse, but only 15% of them (two women) underwent further reconstructive surgery. In the OPTIMAL study, as previously reported, 29 women (19.2%) had symptomatic recurrent prolapse; however, only 17.2% of them (five women) were reoperated. Based on this study, we are not able to explain this difference in approach.

In our study, the overall adverse event rate was 24% (23/95), which included 15% (14/95) women with postoperative urinary tract infection or short-term urine retention. Overall, the rate of serious adverse events was 10% (9/95).

The OPTIMAL study [13] showed a serious adverse event rate as high as 16.5%.

There is currently no consensus on the treatment of patients with apical post-hysterectomy prolapse or apical prolapse in patients with previous POP surgery [6]. A systematic review on this theme from 2017 by Coolen et al. [6] concluded that a comparison of techniques was difficult and a meta-analysis not possible due to the heterogeneity of the different studies on the subject.

Our study, like the OPTIMAL study, demonstrates that the rate of recurrent prolapse associated with HUSLS and the rate of adverse events might be higher than originally described. From this perspective, HULS may not be the optimal first choice of operation for vaginal vault prolapse in patients previously operated due to pelvic organ prolapse.

There are limitations to the present study that should be considered when interpreting the results. First, our data is limited by our relatively small sample size as well as our retrospective, single-institution approach. Second, even though we had a relatively long follow-up period, we did not routinely examine the women beyond the six-month follow-up. This might cause underestimation of the recurrence rate.

Also, the women in our cohort differ from other studies when it comes to the degree of prolapse and the number of previous operations. This may limit the generalisability; however, it may also reflect the real challenges met in the clinic. At the time of these operations, the Pelvic Organ Prolapse (POP-Q) system was not used in our department. Also, it was not noted in the medical charts how many of the women with preoperatively apical stage two prolapse had prolapse beyond the hymen. In general, different grading systems to describe prolapse, as well as different patient characteristics and success criteria, must be kept in mind when comparing the results in the various publications.

## Conclusion

This retrospective study of 95 women with a significant degree of prolapse and a high prevalence of previous pelvic operations demonstrates that the rate of recurrent prolapse associated with HUSLS might be higher than originally described. In conclusion, HUSLS may not be the optimal first choice of operation in this group of patients.

## Data Availability

The datasets generated and analysed during the current study are not publicly available, the study population is small, and Denmark is a small country. With more detailed data, it might be possible to identify individual patients, which is not legal. However anonymised data are available from the corresponding author on reasonable request.

## Abbreviations

HUSLS: High Uterosacral Ligament Suspension
ICS-Q: International Continence Society Quantification System
POP: Pelvic organ prolapse
